# Glutathione *S*-Transferase Pi Prevents Sepsis-Related High Mobility Group Box-1 Protein Translocation and Release

**DOI:** 10.3389/fimmu.2018.00268

**Published:** 2018-02-19

**Authors:** Yi Zhou, Xiang Cao, Yang Yang, Jing Wang, Weidong Yang, Peiling Ben, Lei Shen, Peng Cao, Lan Luo, Zhimin Yin

**Affiliations:** ^1^Jiangsu Province Key Laboratory for Molecular and Medical Biotechnology, College of Life Science, Nanjing Normal University, Nanjing, China; ^2^State Key Laboratory of Pharmaceutical Biotechnology, School of Life Sciences, Nanjing University, Nanjing, China; ^3^Laboratory of Cellular and Molecular Biology, Jiangsu Province Institute of Traditional Chinese Medicine, Nanjing, China

**Keywords:** glutathione *S*-transferases P, phosphorylation, high mobility group box-1 protein, classic protein kinase C, sepsis

## Abstract

Glutathione *S*-transferase Pi (GSTP) was originally identified as one of cytosolic phase II detoxification enzymes and also was considered to function *via* its non-catalytic, ligand-binding activity. We have reported that GSTP played an anti-inflammatory role in macrophages, suggesting that GSTP may have a protective role in inflammation. In this study, we deleted the murine *Gstp* gene cluster and found that GSTP significantly decreased the mortality of experimental sepsis and reduced related serum level of high mobility group box-1 protein (HMGB1). As HMGB1 is the key cytokine involved in septic death, we further studied the effect of GSTP on HMGB1 release. The results demonstrated that a classic protein kinase C (cPKC) dependent phosphorylation of cytoplasmic GSTP at Ser184 occurred in macrophages in response to lipopolysaccharide (LPS) stimulation. Phosphorylated GSTP was then translocated to the nucleus. In the nucleus, GSTP bound to HMGB1 and suppressed LPS-triggered and cPKC-mediated HMGB1 phosphorylation. Consequently, GSTP prevented the translocation of HMGB1 to cytoplasm and release. Our findings provide the new evidence that GSTP inhibited HMGB1 release *via* binding to HMGB1 in the nucleus independent of its transferase activity. cPKC-mediated GSTP phosphorylation was essential for GSTP to translocate from cytoplasm to nucleus. To our knowledge, we are the first to report that nuclear GSTP functions as a negative regulator to control HMGB1 release from macrophages and decreases the mortality of sepsis.

## Introduction

Glutathione *S*-transferases (GSTs) refer to a group of cytosolic phase II detoxification enzymes, which have both a GSH-binding site and a substrate-binding site (H-site) that catalyze the nucleophilic attack of the sulfur atom of glutathione (GSH) on electrophilic groups of substrate molecules (1, 2). Glutathione *S*-transferase Pi (GSTP) is the most abundant member of cytosolic GSTs in the mammalian cells (2), and Tyr-7 in GSTP has been considered to be necessary for its enzyme activity (3, 4). GSTP also functions *via* its non-catalytic ligand-binding activity. It has been reported that GSTP interacts with JNK, TRAF2, and STAT3 and involves in protecting cells against apoptosis, oxidative stress, and angiotensin II stimulation (5–8). In tumor cell lines, GSTP has been identified as a significant factor in carcinogenesis and development of drug resistance (9). We have found that GSTP played an anti-inflammatory role in mouse macrophage-like RAW 264.7 cells and treating mice with recombinant GSTP protein reduced mortality rate of endotoxin shocked mouse (10, 11). Furthermore, a recent report showed that knockdown of GSTP enhanced NF-κB nuclear translocation, transcriptional activity, and pro-inflammatory cytokine production in response to lypopolysaccharide (LPS) (12). These findings suggested that cellular GSTP might function as an anti-inflammatory factor for preventing the progression of bacteremia or sepsis.

Sepsis, a systemic inflammatory response induced by aggressive infection, is a leading cause of death in intensive care units associated with significant public healthcare problem (13, 14). The mortality in sepsis is mostly attributed to multiple organ dysfunction due to a dysregulated host response to infection (15, 16). Although some interventions have improved the outcomes of septic patients, severe sepsis still carries a high mortality rate. The persistently high mortality rate suggests a need for novel therapeutic interventions to improve survival, requiring thorough understanding of sepsis pathophysiology (17). The theory about sepsis pathophysiology with a stronger experimental support is the exaggeration of inflammatory response. The positive feedback loop between cytokines and immune cells highly activate host immune system and lead to uncontrolled proinflammatory responses (14, 18, 19). The animal sepsis models including LPS injection, bacterial injection, and cecal ligation and puncture (CLP) have recapitulated this process. However, treatment of sepsis based on anti-tumor necrosis factor (TNF)-α antibody and interleukin (IL)-1 receptor antagonist did not demonstrate any clinical benefit (20–22).

Immune cells recognize not only microorganisms (pathogen-associated molecular patterns; PAMPs) but also damaged tissues (damage-associated molecular patterns; DAMPs) (19). High mobility group box-1 protein (HMGB1), an important DAMP molecule, has been demonstrated to be a late pro-inflammatory mediator of sepsis. HMGB1 was originally known as an abundant and chromosomal protein, which is involved in DNA replication, repair, and transcription (23, 24). Macrophages and monocytes actively secrete HMGB1 after being challenged by different stimulators, such as LPS, TNF-α or IL-1 (25–27). Activation of macrophages results in the redistribution of HMGB1 from the nucleus to cytoplasm, followed by the release *via* an unconventional protein secretion pathway (25, 27, 28). The modification of HMGB1, such as phosphorylation and acetylation, is vital for its translocation (25, 29–32). Released into the extracellular space, HMGB1 in turn stimulates production of massive inflammatory cytokines, which further expand the inflammatory response (33, 34). One notable point is that as a late-phase cytokine, the increase of HMGB1 release is tightly associated with increased mortality in animal models of sepsis (23, 35–37). Clinically, enhanced blood HMGB1 levels are detectable in septic patients up to 7 days after a diagnosis of sepsis, and these HMGB1 concentrations are related with the severity of organ damage and death (38). Since it has emerged as a key mediator in sepsis and contributes to the high lethality of sepsis, HMGB1-targeting strategies may be effective to treat sepsis (39).

Here, we report an important role of GSTP in inhibiting sepsis development by using *Gstp*^−/−^ mice and cecal ligation and puncture (CLP) sepsis model. More importantly, we discovered the novel mechanism that LPS stimulation triggered a classic protein kinase C (cPKC)-dependent phosphorylation of GSTP and translocation of GSTP from cytoplasm to nucleus. In the nucleus, GSTP bound to HMGB1 and consequently prevented HMGB1 to translocate to the cytoplasm and release from macrophages. Our research suggested a novel mechanism through which GSTP negatively regulated sepsis development.

## Materials and Methods

### Construction and Identification of *Gstp* Knockout Mice

*Gstp* knockout mice were purchased from ViewSolid Biotech (Beijing, China). Clustered regularly interspaced short palindromic repeats/CRISPR-associated 9 (CRISPR/Cas9) systems was used to disrupt both *Gstp* genes in mice. The DNA of *Gstp* knockout mice was extracted and subjected to PCR by using MightyAmp™ Genotyping Kit (Takara, Ohtsu, Japan), which as shown in Figure S1C in Supplementary Material. The following primers were used: forward, 5-CAAGGCTCTACTTCCCTCACCATG-3 and reverse, 5-ACAGGAAAGGAAACTAGGGAGG-3.

### CLP-Induced Sepsis Animal Model

Sepsis was induced in 7- to 8-week-old male C57BL/6 *Gstp*^−/−^ or wild-type mice (18–20 g) by CLP. Mice were anesthetized with sodium pentobarbital (30 mg/kg), and the severity of sepsis was highly rested with the extent of cecal ligation. High-grade sepsis was used in our experiments as described (40), which resulted in survival rates of ~25%. The sham-operation which included the same procedure except for ligation and perforation of the cecum was conducted on control mice. Then, mice were administered intraperitoneally with 2 mg/kg of GSTP (WT), or sterile saline, respectively, 30 min after CLP surgery. The survival rates of mice were monitored up to 72 h. In parallel experiments, blood samples were collected for HMGB1 detection 12 h after CLP, and lungs were excised from mice 10 h after CLP for histological analysis by hematoxylin eosin staining. The *in vivo* study was approved by Medical Laboratory Animal Research Institute of Medical Sciences China [Permit Number: SYXK (Jing) 2014-0004]. All treatments of mice in this study were in strict agreement with guidelines on ARRIVE and recommendations from an NIH-sponsored workshop regarding experimental design and reporting standards (41).

### Cell Culture and Transfection

RAW264.7 murine macrophage-like cells and THP-1 cells (human acute monocytic leukemia cell line) were obtained from the CBCAS (Cell Bank of the Chinese Academic of Sciences, Shanghai, China). HEK293 (human embryonic kidney) cells were purchased from Institute of Biochemistry and Cell Biology (Chinese Academy of Sciences, Shanghai, China). RAW264.7 and HEK293 cells were cultured in DMEM (Wisent) supplemented with 10% fetal bovine serum. THP-1 cells were cultured in RPMI 1640 (Wisent) with 10% (v/v) fetal bovine serum (Wisent). These cell lines were maintained at 37°C in an atmosphere of 5% CO_2_. X-tremeGENE HP DNA Transfection Reagent (Roche) was used for transient transfection, and the total amount of transfected DNA was normalized by addition of empty control plasmids.

### Antibodies and Reagents

High mobility group box-1 protein monoclonal antibody (mAb) was from R&D (Minneapolis, MN, USA). GSTP, cPKC, chromosomal region maintenance 1 (CRM1), and phospho-Serine mAb were from Abcam (Cambridge, United Kingdom). Polyclonal antibodies against β-actin, laminB, phospho-GSTP (Ser184), His-tag, and Flag-tag were from Bioworld Biotechnology (Minneapolis, MN, USA). Rabbit monoclonal antibodies against phospho-Threonine and mouse mAb against phospho-Tyrosine were obtained from Cell Signaling Technology (Beverly, MA, USA). Secondary antibodies coupled to IRDye800 flurophore were purchased from Rockland (Gilbertsville, PA, USA). LPS (from Escherichia coli 0111:B4), phorbol-12-myristate-13-acetate (PMA; TPA, Phorbol 12-myristate 13-acetate), and ATP (adenosine triphosphate) were from Sigma-Aldrich (St. Louis, MO, USA). 6-(7-nitro-2,1,3-Benzoxadiazol-4-ylthio)-hexanol (NBDHEX) was synthesized as previously reported and dissolved in DMSO. The broad-spectrum PKC inhibitor Gö6983, the PKA inhibitor H 89 2HCl, and the cPKC inhibitor GF109203X were purchased from Selleckchem.

### DNA Constructs

As shown in Figure S4B in Supplementary Material, pcDNA3.1-Flag-GSTP, pcDNA3.1-Flag-HMGB1, pcDNA3.1-Flag-GSTM, six GSTP phosphorylation mutants including pcDNA3.1-Flag-GSTP-S42A (in which serine-42 was mutated to alanine, a S42 non-phosphorylatable mutant), pcDNA3.1-Flag-GSTP-S42D (in which serine-42 was mutated to aspartate, a Ser42 constant-phosphomimetic mutant), pcDNA3.1-Flag-GSTP-S184A (in which serine-184 was mutated to alanine, a Ser184 non-phosphorylatable mutant), pcDNA3.1-Flag-GSTP-S184D (in which serine-184 was mutated to aspartate, a Ser184 constant-phosphomimetic mutant), pcDNA3.1-Flag-GSTP-Y198F (in which tyrosine-198 was mutated to phenylalanine, a Tyr198 non-phosphorylatable mutant) and pcDNA3.1-Flag-GSTP-Y198D (in which tyrosine-198 was mutated to aspartate, a Tyr198 constant-phosphomimetic mutant), pET28a-HMGB1, pET28a-GSTP(WT), pET28a-GSTP(S184A), pET28a-GSTP(S184D) were constructed by using molecular cloning technology. DNA fragment encoding wild-type HMGB1, Flag-tagged GSTP, Flag-tagged GSTM, Flag-tagged HMGB1 were produced by high-fidelity PCR, and then cloned into pcDNA3.1 or pET28a vector. Over-lap PCR was used to generate six GSTP phosphorylation-mimicking mutants (42). All of the plasmids were confirmed using DNA sequencing.

### Co-Immunoprecipitation and Immunoblotting Analysis

Cells were washed twice with phosphate-buffered saline (PBS), and then lysed in a lysis buffer on ice for 30 min, and then cell lysates were centrifuged at 12,500 rpm for 15 min. Samples were immunoprecipitated with indicated antibodies and protein A/G plus-agarose beads (Santa Cruz Biotechnology) at 4°C overnight and washed four times with the lysis buffer. After resolved in SDS-PAGE, the immunoprecipitates were transferred onto polyvinylidene fluoride membranes (Whatman, GE Healthcare, NJ, USA). Then the membrane was blocked with 5% BSA for 1 h at room temperature, followed by incubating with primary antibodies and IRDye800 flurophore-conjugated antibodies (LI-COR Biosciences, Lincoln, NE, USA). LI-COR Odyssey Infrared Imaging System was used to visualize the protein complexes.

### Immunofluorescence Microscopy

Cells were rinsed two times with PBS followed by 4% paraformaldehyde fixation for 30 min and permeabilizing with 0.2% Triton X-100 for 20 min. After being blocked by 5% BSA to reduce non-specific binding, the cells were incubated with primary antibodies overnight. After washing (0.1% Tween-20 in PBS), cells were incubated with FITC-conjugated secondary antibody (Invitrogen, Carlsbad, CA, USA) for 1 h. DAPI (4,6-diamidino-2-phenylindole) (Invitrogen, Carlsbad, CA, USA) was used to show the nuclei. Slides were mounted and examined under Nikon A1 confocal laser microscope system (Tokyo, Japan).

### Nuclear and Cytoplasmic Extract Preparation

Nuclear and cytoplasmic extracts were prepared by using NE-PER nuclear and cytoplasmic extraction Kit (Thermo Scientific, Rockford, IL, USA) according to the instructions specified by the manufacturer. In brief, cells were harvested by centrifuging at 500 *g* for 5 min, followed by washing with PBS. Cells were then lysed in ice-cold cytoplasm extraction reagent I (CER I) for 10 min and ice-cold CER II for 1 min. Cytoplasmic component was extracted by centrifuging at 16,000 *g* for 5 min. Proteins of the nuclear material were then extracted by adding nuclear extraction reagent to the insoluble fraction with vigorous vortex per 10 min for four times and centrifugation at 16,000 *g*. The extracts were stored at −80°C until use.

### Cytokine Measurement

Mouse serum samples were obtained by centrifuging mouse blood at 3,000 rpm for 15 min. For cultured cells, the cultural medium samples were collected. The levels of HMGB1 in the mouse serum and cultural medium were determined by using the quantitative HMGB1 ELISA kit (Shanghai Westang Bio-Tech Co., Ltd.), according to manufacturer’s instructions and standard curves of recombinant HMGB1.

### Realtime-PCR Analysis

High Pure RNA Isolation Kit (Roche, Germany) was used to extract total RNA. cDNA was synthesized using PrimeScript reverse transcription reagents (TaKaRa, Shiga, Japan). Real-time PCR was carried out on a LightCycler (Roche, Germany) using SYBR Premix EX Taq reagent (TaKaRa, Shiga, Japan). The mouse *HMGB1* transcript was amplified using the following primers: forward, 5-CGGATGCTTCTGTCAACTTCT-3 and reverse, 5-AGTTTCTTCGCAACATCACCA-3. Mouse β*-actin* transcript: forward primer, 5-CATCCGTAAAGACCTCTATGCCAA-3 and reverse, 5-ATGGAGCCACCGATCCAC-3. The housekeeping gene, β*-actin*, was used to normalize tested genes, and data quantification was performed using the ΔΔCT method.

### GSTP Catalytic Activity Analysis

Cells were harvested by ultrasonic treatment, and the GSTP enzymatic activity was determined by using glutathione *S*-transferase (GSH-ST) kit (Nanjing Jiancheng Bioengineering Institute, Nanjing, China). The reactions were terminated, and the enzyme kinetics of the conjugation of 1-chloro-2,4-dinitrobenzene to GSH was determined as previously described (43).

### Histological Examination

Lung tissues were collected and fixed in 4% paraformaldehyde, then embedded in paraffin using standard procedures, following by serially sectioned as previously described (44). Tissue sections were stained with Hematoxylin eosin and were observed under a light microscope (Olimpus, Japan). Lung injuries were evaluated by infiltration of inflammatory cells, to grade the degree of lung injury in four fields. Each category was scored according to the following system (0 = normal; 1<25%; 2 = 25–50%; 3 = 50–75%; 4>75%) (45).

### *In Vitro* Pull-Down Binding Assays

To determine whether GSTP binds directly to HMGB1, recombinant polyhistidine-tagged GSTP (WT), GSTP (S184A), GSTP (S184D), and HMGB1 proteins were expressed in *Escherichia coli* BL21 (DE3) and purified through Ni-NTA spin column (Qiagen). Flag-HMGB1 or Flag-GSTP (WT), GSTP (S184A), and GSTP (S184D) were transfected into HEK293 cells, and then, the lysates were incubated with His-GSTP or His-HMGB1 immobilized on Ni^2+^–IDA–agarose beads (Novagen) in the lysis buffer at 4°C for 8 h. Immunoblotting was performed to analyze the pull-down interests.

### GSTP Preparation for MALDI-QIT-TOF-MS Analysis

RAW264.7 cells were transfected with Flag-GSTP and then were treated with LPS (500 ng/ml) for 30 min. Cell lysates were immunoprecipitated with anti-Flag antibody, run in SDS/PAGE gel, and stained with Coomassie Brilliant Blue. Gels were cut into pieces, subjected to trypsin (sequencing grade; Promega Corporation, Madison, WI, USA) digestion according to the manufacturer’s instructions for phosphorylation modification analysis, which using a Titansphere Phos-TiO kit (GL Sciences, Tokyo, Japan), and desalted using ZipTip C18 pipette tips (Millipore, Temecula, CA, USA). Characterization of phospho-peptides were determined by MALDI-QIT-TOF with an AXIMA Resonance (Shimadzu/Kratos, Kyoto, Japan), and a subsequent database search was performed with MASCOT search engines (Matrix Science, Boston, MA, USA).

### Statistical Analysis

The experimental data acquired from cells and mice one-way ANOVA and shown as mean ± SD. Kaplan–Meier survival analysis with the log-rank test for between-group comparisons was used to analyze the survival rates of CLP mice. Western blotting analyses were repeated three times with similar trends and quantified using ImageJ (46). Statistical analysis was carried out using the SPSS 13.0 software (SPSS Inc., Chicago, IL, USA). A value of *P* < 0.05 was considered significant.

## Results

### GSTP Improves Survival and Reduces Serum HMGB1 Level in Septic Mice

**Figure 1:**
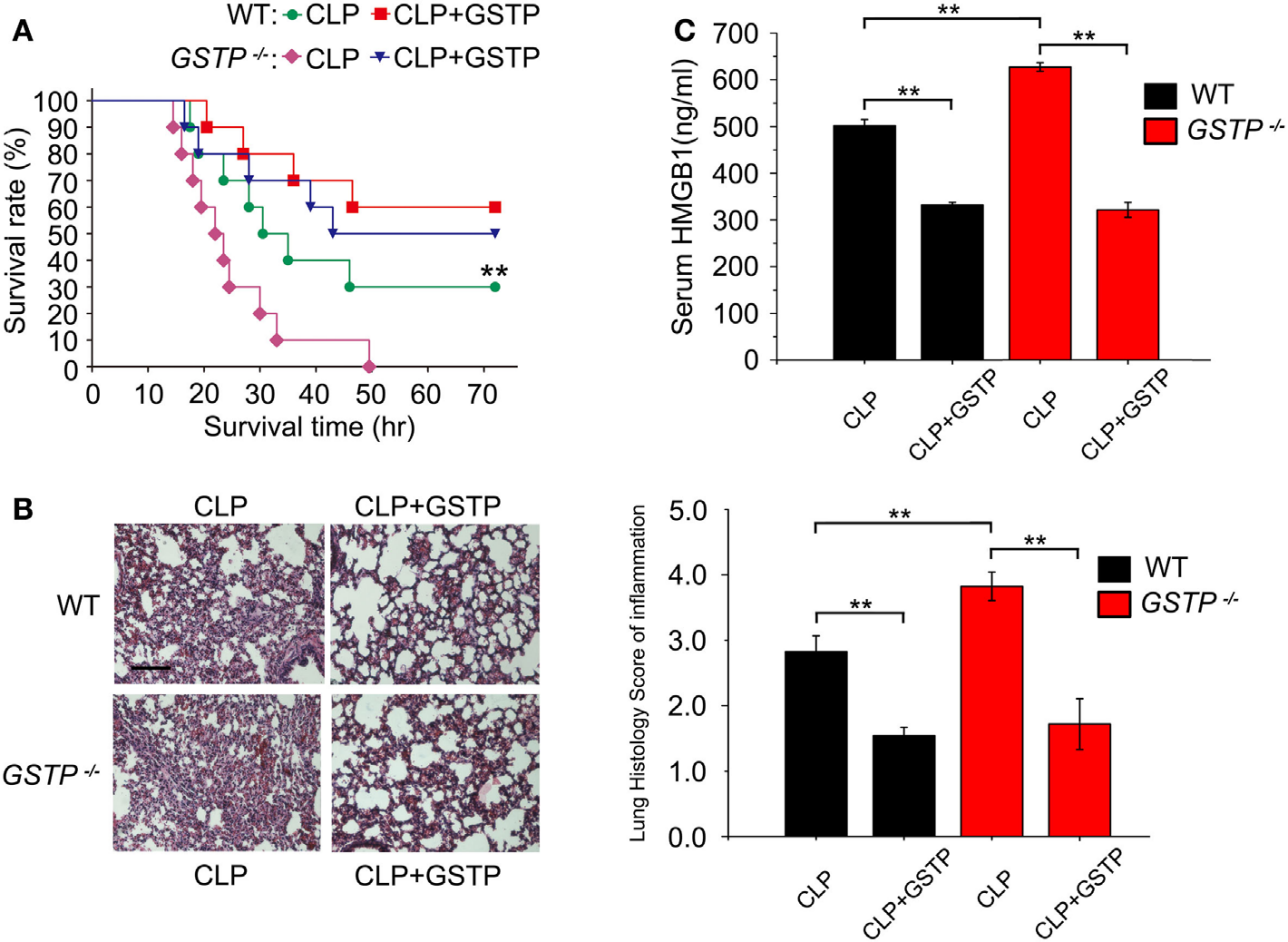
Glutathione *S*-transferase Pi (GSTP) improves survival and reduces serum high mobility group box-1 protein (HMGB1) level in septic mice. **(A)** *Gstp*^−/−^ and wild type C57BL/6 mice were treated with 2 mg/kg of GSTP or lypopolysaccharide-free saline, respectively, 30 min after CLP surgery, and survival rate of mice was monitored continuously. **(B)** Lung tissues of the mice were collected and subjected to Hematoxylin and Eosin staining 10 h after CLP, and examined by light microscopy (magnification, ×100). Scale bar: 100 µm. Histological scoring of lung injury was evaluated based on inflammatory infiltrate in CLP mice. **(C)** Detection of mouse serum HMGB1 level by ELISA. Mouse blood was collected from retroorbital sinuses of mice. Serum HMGB1 level was measured by ELISA 12 h after CLP surgery. Data information: in **(A)**, Kaplan–Meier analysis was used to analyze the survival rates of CLP mice. *n* = 10 mice/group. **P<0.01 versus *Gstp*^−/−^ CLP group by the log-rank test for between-group comparisons. In **(B,C)**, Graphs show mean ± SD. *n* = 8–10 mice/group. **P<0.01 versus corresponding CLP group by unpaired Student’s *t*-test. Data shown were representative of three independent experiments.

CLP model is the most frequently used experimental sepsis model, which simulates a systemic inflammatory response and multiple organ dysfunction in human sepsis (40, 47). HMGB1 stimulates inflammatory cytokine production and creates an inflammatory cascade, which tightly associates with increased mortality of sepsis (48, 49). To evaluate the role of GSTP in sepsis pathophysiology, we deleted the mouse *Gstp* gene cluster. As shown in Figures S1A,B in Supplementary Material, *Gstp* genes lie on chromosome 19, and there were large fragment deletions and frameshift mutations in the mouse *Gstp* genes. The agarose gel electrophoresis and DNA sequence analysis showed that the PCR products were about 900 bp (Figure S1C in Supplementary Material) and there was 4,314 bp DNA sequence deletion in the *Gstp* knockout mouse. The immunoblotting showed that there was no GSTP protein signal in macrophages from *Gstp*^−/−^ mice (Figure S1D in Supplementary Material). We found that *Gstp*^−/−^ mice displayed a higher mortality rate, severer acute lung injury and higher HMGB1 release level than wild type C57BL/6 mice after CLP surgery (Figures 1A–C), which first demonstrated that GSTP acted as a key negative regulator in sepsis development. Administration of GSTP recombinant protein (2 mg/kg, i.p.) to both wild type and *Gstp*^−/−^ mice 30 min after CLP surgery significantly alleviated CLP-induced acute lung injury, decreased HMGB1 release and mortality of mice (Figures 1A–C). Meanwhile, according to the lung histology score of inflammation, GSTP remarkably reduced the inflammatory infiltrate increased in CLP mice, which might lead to the decrease of the higher level of HMGB1 found in the serum and mortality of mice (Figures 1A–C). These findings not only supported the importance of endogenous GSTP in protecting against excessive inflammation but also the potential therapeutic effects of recombinant GSTP protein on sepsis.

### GSTP Inhibits Nuclear-Cytoplasmic Translocation of HMGB1 and Release from Macrophages

**Figure 2:**
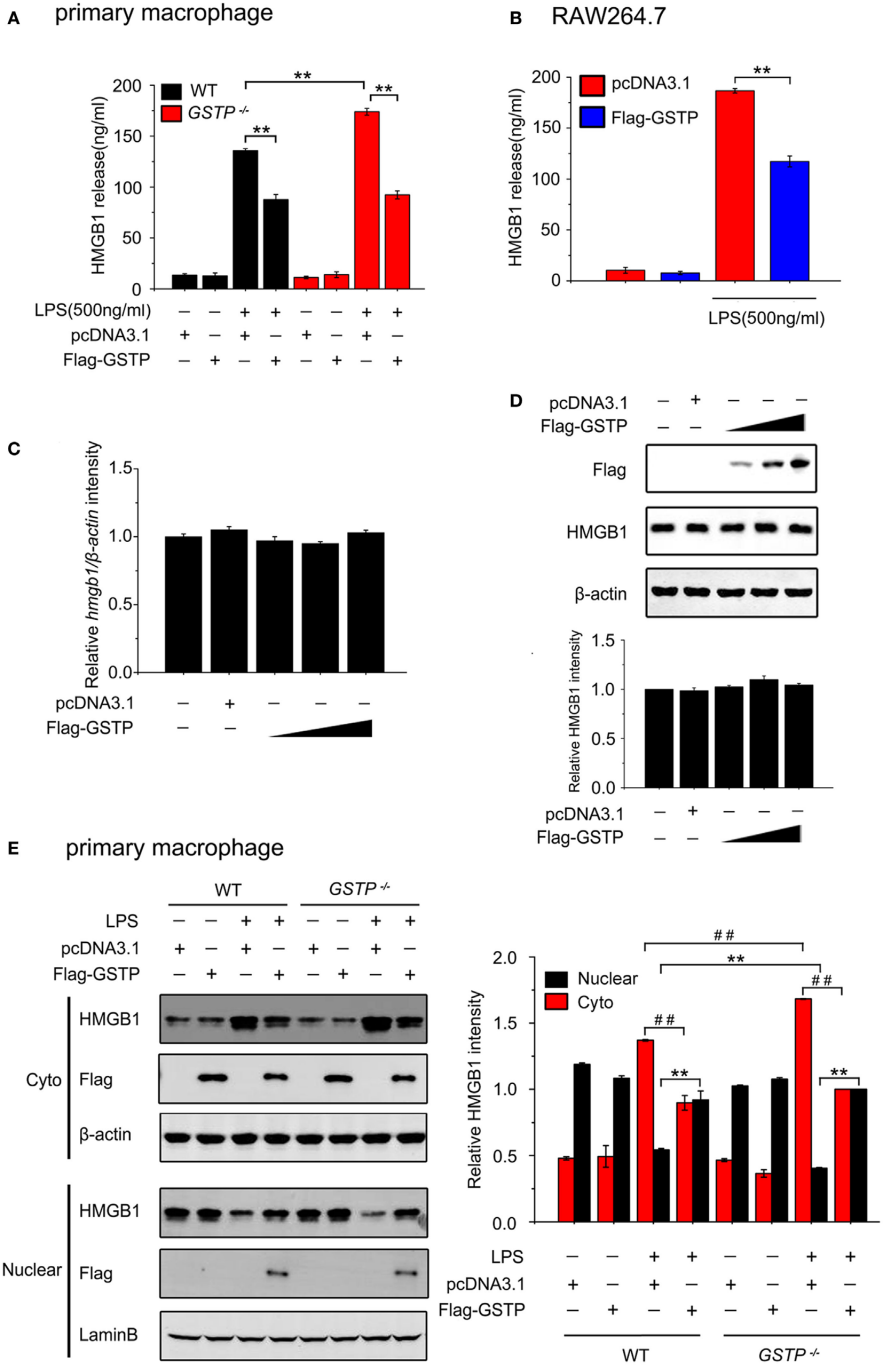

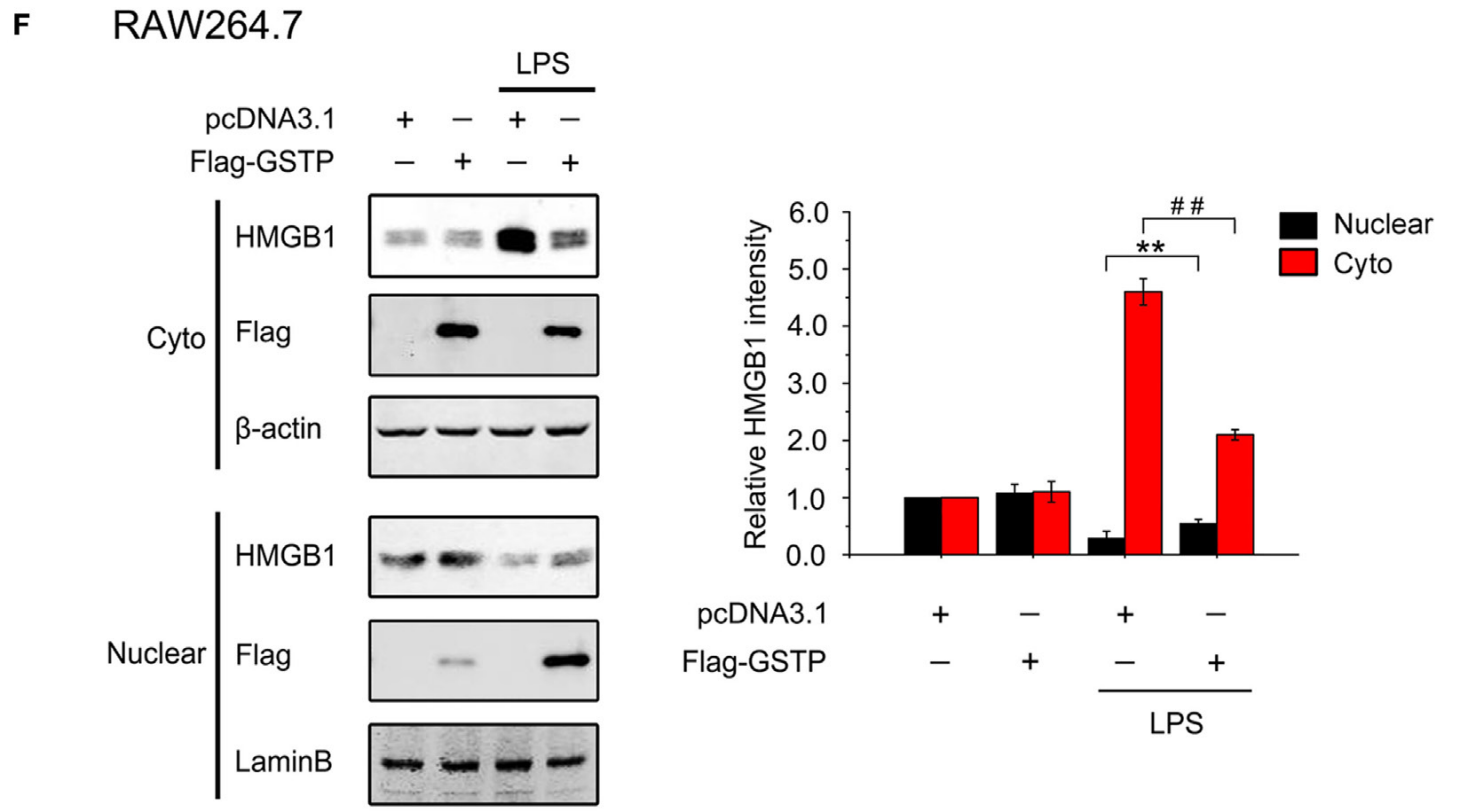
Glutathione *S*-transferase Pi (GSTP) inhibits lypopolysaccharide (LPS)-induced high mobility group box-1 protein (HMGB1) cytoplasm translocation and release from macrophages. **(A,B)** Primary peritoneal macrophages **(A)** and RAW264.7 cells **(B)** were transfected with Flag-GSTP or pcDNA3.1 (control) and then were stimulated with or without LPS (500 ng/ml). After 18 h, the levels of HMGB1 in the culture medium were measured by ELISA. **(C,D)** RAW264.7 cells were transfected with Flag-GSTP (1, 1.5 and 2 µg), or empty vector. **(C)** Quantifications of *HMGB1* transcripts were determined by real-time PCR and β*-actin* was used as internal control. **(D)** Cell lysates were subjected to Western blot with antibodies against HMGB1 and Flag. **(E,F)** Cells were treated as same as in A and B, and 6 h after LPS stimulation, nuclear and cytoplasmic fractions were subjected to Western blot using HMGB1 and Flag antibodies. Lamin B and β-actin were detected to show equal loading nuclear and cytoplasmic proteins. Data shown were representative of three independent experiments. Data information: in **(A,B)**, data are presented as mean ± SD. **P<0.01 versus corresponding LPS-treated group by unpaired Student’s *t*-test. In **(E,F)**, data are presented as mean ± SD. **P<0.01 versus corresponding LPS-treated nuclear group by unpaired Student’s *t*-test. ^##^*P*<0.01 versus corresponding LPS-treated cytoplasmic group by unpaired Student’s *t*-test.

To investigate the effect of GSTP on release of HMGB1 from macrophages, we performed *in vitro* experiments on cultured primary cells and cell lines. Primary mouse peritoneal macrophages, mouse macrophage-like RAW 264.7 cells, and human monocytic THP-1 cells were transfected with GSTP encoding expression vector (pcDNA3.1-Flag-GSTP) or empty vector (pcDNA3.1), followed by stimulation of LPS (500 ng/ml) for 18 h, and the levels of HMGB1 in the culture medium were determined by ELISA. The results demonstrated that LPS induced HMGB1 release from all types of cells (Figures 2A,B; Figure S2A in Supplementary Material). The primary peritoneal macrophages from the *Gstp*^−/−^ mice showed a higher HMGB1 release level than that from wild type C57BL/6 mice (Figure 2A). However, LPS-induced HMGB1 release significantly reduced in cells transfected with GSTP (Figures 2A,B; Figure S2A in Supplementary Material). However GSTP did not affect HMGB1 expression at mRNA and protein levels (Figures 2C,D). As shown in Figures 2E,F and S2B in Supplementary Material, HMGB1 was predominantly localized in the nucleus of unstimulated cells but was detected in cytoplasmic compartment 6 h after of LPS stimulation. The cytoplasmic level of HMGB1 in primary peritoneal macrophages from *Gstp*^−/−^ mice was higher than that from wild type mice (Figures 2E,F; Figure S2B in Supplementary Material). When these cells were transfected with GSTP the cytoplasmic HMGB1 level reduced (Figures 2E,F; Figure S2B in Supplementary Material). In comparison, LPS-stimulated cytoplasmic translocation and release of HMGB1 was not affected by GSTM, another member of GSTs (Figures S2C,D in Supplementary Material). These results strongly suggested that GSTP attenuated HMGB1 release through suppressing its cytoplasm translocation rather than the protein expression.

### The Effect of GSTP on HMGB1 Release Is Independent of Its Transferase Activity

**Figure 3:**
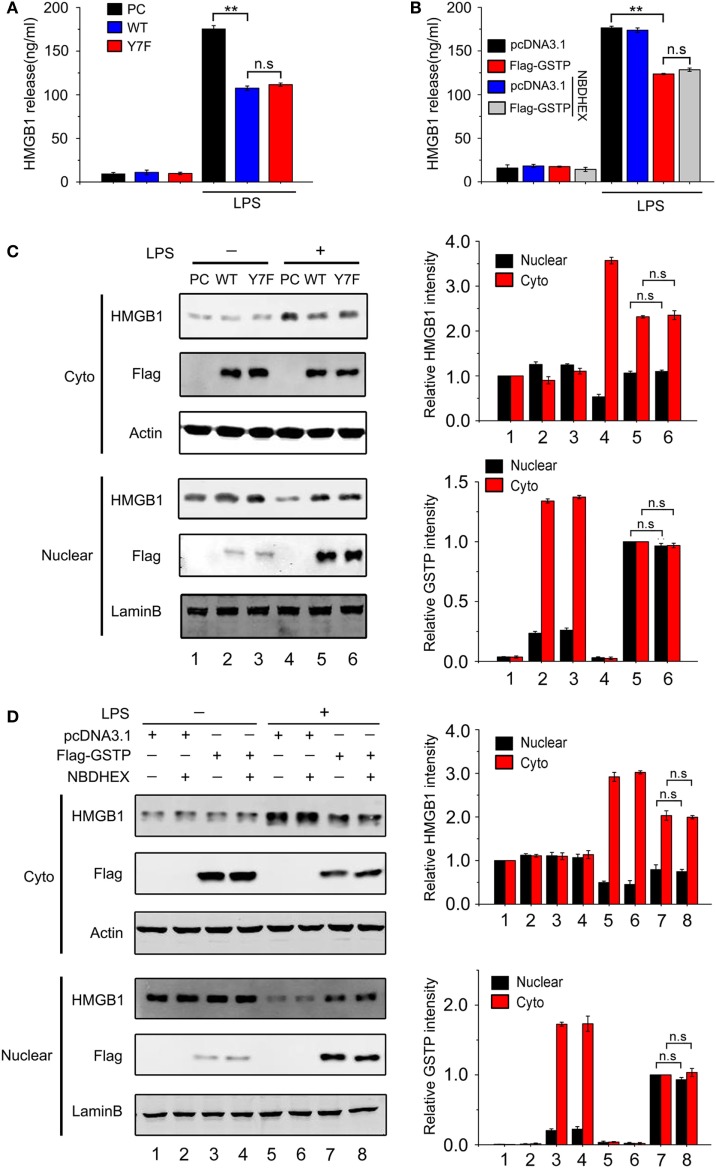
The inhibitory effect of glutathione *S*-transferase Pi (GSTP) on high mobility group box-1 protein (HMGB1) release is enzymatic activity independent. **(A)** RAW264.7 cells were transfected with Flag-GSTP(WT), Flag-GSTP(Y7F), or empty vector and then stimulated with or without lypopolysaccharide (LPS; 500 ng/ml) for 18 h. HMGB1 levels in the culture medium were measured by ELISA. **(B)** Flag-GSTP(WT) or empty vector was transfected into RAW264.7 cells, and then cells were pre-treated with 6-(7-nitro-2,1,3-Benzoxadiazol-4-ylthio)-hexanol for 2 h, followed by LPS (500 ng/ml) treatment for 18 h. HMGB1 levels the culture medium were detected by ELISA. **(C,D)** Cells were treated as same as in **(A,B)**, nuclear and cytoplasmic fractions were analyzed by Western blot using HMGB1 and Flag antibodies. Equal loading protein was confirmed by detecting nuclear lamin B and cytoplasmic GAPDH. Data information: In **(B–D)**, data are presented as mean ± SD. **P<0.01; n.s., not significant (*P* > 0.05), versus corresponding LPS-treated group by unpaired Student’s *t*-test.

As GSTP is an enzyme involved in phase II metabolism (1), we next analyzed if the inhibitory effect of GSTP on HMGB1 release was related with its catalytic activity. RAW264.7 cells were transfected with pcDNA3.1, pcDNA3.1-Flag-GSTP (WT, wild type), or the catalytically inactive mutant pcDNA3.1-Flag-GSTP (Y7F, phenylalanine replaced tyrosine in the seventh amino-terminal position), respectively. 18 h after LPS stimulation, HMGB1 levels in the culture media were detected by ELISA. The result showed that there was no difference in HMGB1 level between GSTP (Y7F) and GSTP (WT) transfected cells (Figure 3A). NBDHEX is an inhibitor for GSTs. As shown in Figures S3A,B in Supplementary Material, NBDHEX inhibited GSTP enzymatic activity but exhibited no cytotoxity at 0.5 µM concentration. RAW264.7 cells transfected with pcDNA3.1 or pcDNA3.1-Flag-GSTP were pretreated with NBDHEX (0.5 µM) for 2 h followed by stimulation with LPS. The effect of GSTP on LPS-induced HMGB1 release was not influenced by NBDHEX (Figure 3B). As shown in Figure 3C, GSTP (Y7F) and GSTP (WT) equally restrained LPS-induced HMGB1 cytoplasmic translocation in RAW264.7 cells and NBDHEX (Figure 3D) also did not inhibit such effect of GSTP. Altogether, preceding results suggested that the inhibitory effects of GSTP on HMGB1 release and cytoplasmic translocation were independent of its enzymatic activity.

### GSTP Enters the Nucleus and Interacts with HMGB1 in Response to LPS Stimulation

**Figure 4:**
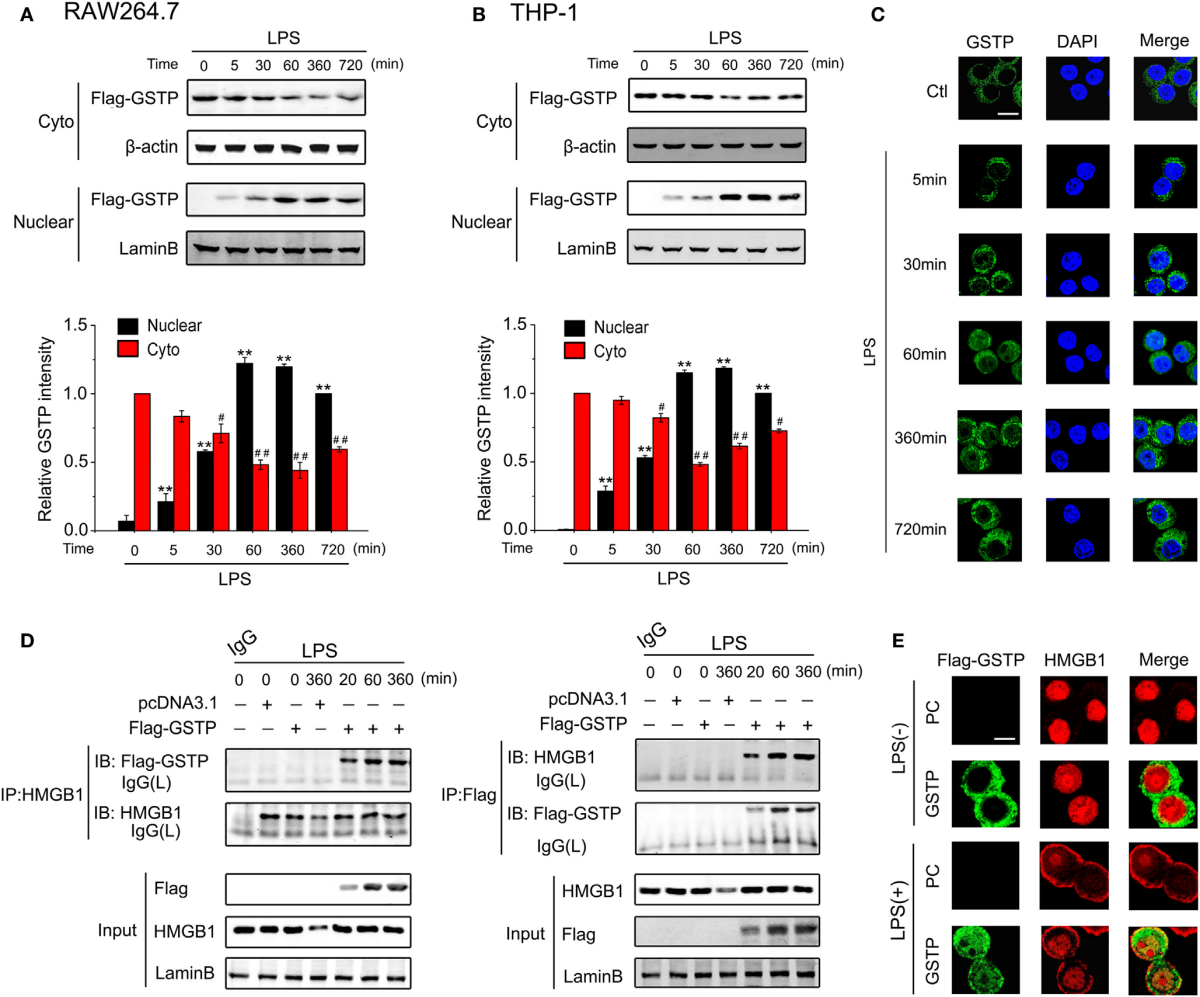
Glutathione *S*-transferase Pi (GSTP) enters the nucleus and interacts with high mobility group box-1 protein (HMGB1) in response to lypopolysaccharide (LPS) stimulation. **(A,B)** RAW264.7 cells **(A)** and THP-1 cells **(B)** were transfected with Flag-GSTP. Cells were then treated with LPS (500 ng/ml) for indicated hours. Nuclear and cytoplasmic fractions were subjected to Western blot using the anti-Flag antibody. **(C)** RAW264.7 cells were stimulated with LPS (500 ng/ml) for indicated time, and then, were incubated with anti-GSTP antibody, followed by incubating with Alexa flour488-conjugated secondary antibody (green). The nuclei were counterstained with DAPI (blue). Nuclear and cytoplasmic GSTP were observed under a confocal laser microscope. Scale bar: 10 µm. **(D)** RAW264.7 cells were transfected with Flag-GSTP or empty vector, and then, were stimulated with LPS (500 ng/ml) for indicated time. Nuclear extracts were subjected to immunoprecipitation with anti-HMGB1 antibody (left) or anti-Flag antibody (right) followed by immunoblotting with anti-Flag or anti-HMGB1 antibody. The whole nuclear fractions were immunoblot analyzed with anti-Flag, anti-HMGB1 and anti-Lamin B antibodies. **(E)** After being treated with LPS (500 ng/ml) for 8 h, cells were incubated with rabbit anti-Flag and mouse anti-HMGB1 antibodies, and then, incubated with Alexa flour488-conjugated anti-rabbit (green) and Alexa flour555-conjugated anti-mouse (red) secondary antibodies. The subcellular localization of Flag-GSTP and HMGB1 were examined by confocal microscopy. Scale bar: 10 µm. Data information: in **(A,B)**, data are presented as mean ± SD. **P<0.01 versus untreated nuclear group by unpaired Student’s *t*-test. ^#^*P*<0.05; ^##^*P*<0.01 versus untreated cytoplasmic group by unpaired Student’s *t* test.

The immunoblotting analysis and confocal microscopy observation showed that nuclear GSTP protein level can be detected within 5 min after LPS treatment and peaked at 1 h (Figures 4A–C). The high level of nuclear GSTP was maintained from 1 to 6 h after LPS challenge and just decreased slightly at 12 h (Figures 4A–C). These data indicated that LPS stimulation result in the distribution of GSTP in the nucleus. To analyze how nuclear GSTP interacted with HMGB1, RAW264.7 cells were transfected with Flag-GSTP and then were stimulated with LPS. The nuclear extracts were subjected to immunoprecipitation by using anti-HMGB1 antibody (Figure 4D, left panel) or anti-Flag antibody (Figure 4D, right panel). The complex of GSTP and HMGB1 appeared 20 min after LPS stimulation, increased to the maximum at 1 h and maintained at the high level up to 6 h (Figure 4D). The co-localization of GSTP and HMGB1 in the nucleus was confirmed by the confocal microscopy (Figure 4E). Taken together, these results indicated that in response to LPS stimulation, cytoplasmic GSTP translocated to the nucleus, where it bound to HMGB1.

### Phosphorylation at Serine-184 of GSTP Is Crucial for Its Nuclear Translocation and Interaction with HMGB1

**Figure 5:**
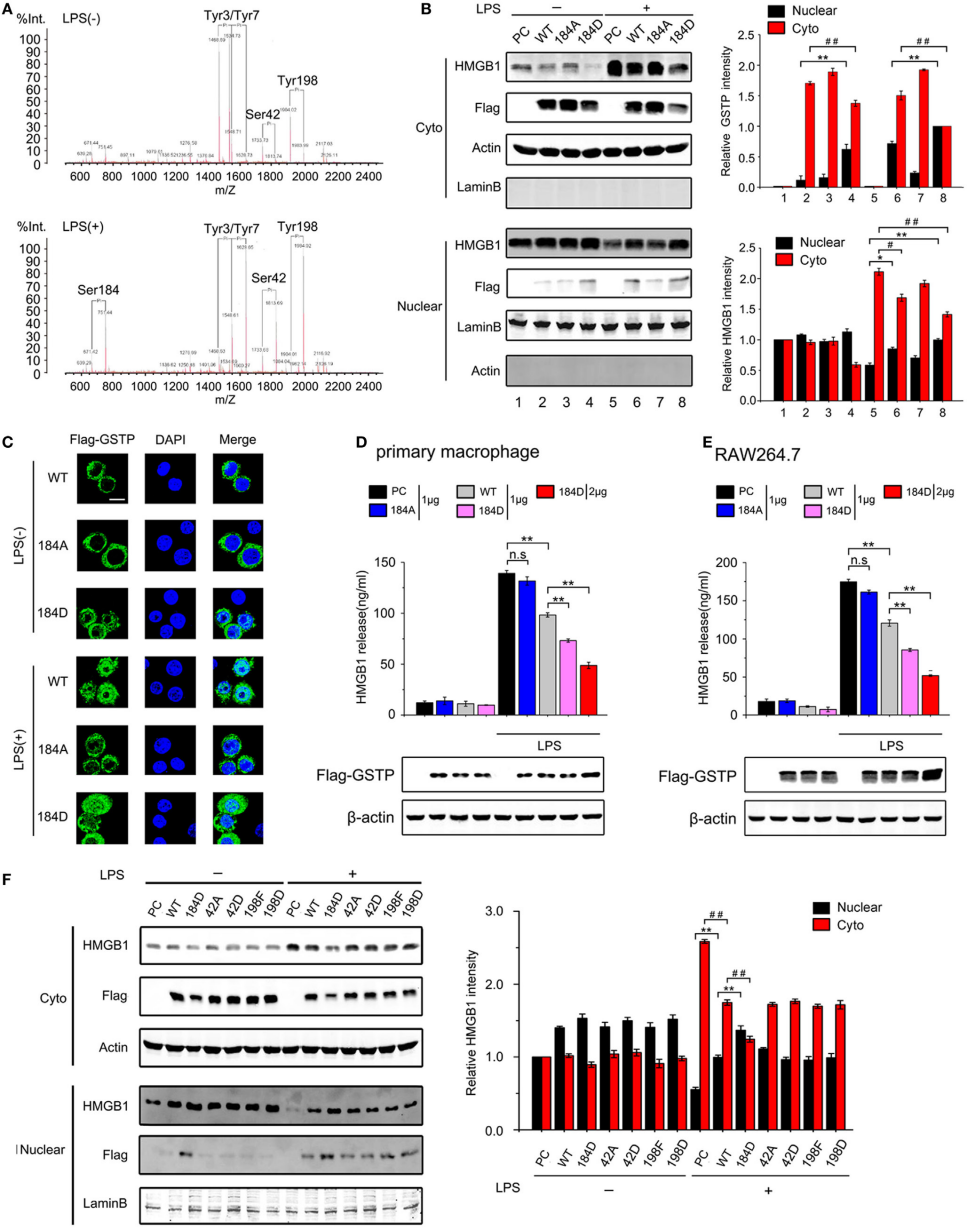

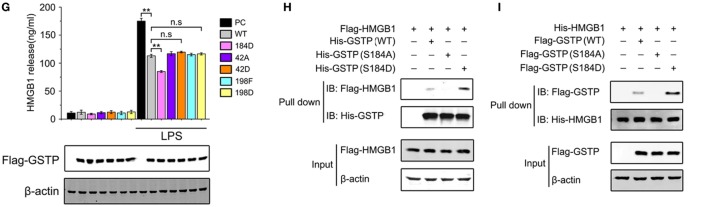
Glutathione *S*-transferase Pi (GSTP) phosphorylation at serine-184 is crucial for its nucleus translocation and interaction with high mobility group box-1 protein (HMGB1). **(A)** The phosphorylation profiles of GSTP in RAW264.7 cells untreated (upper) and treated (bottom) with lypopolysaccharide (LPS; 500 ng/ml) for 30 min were detected by MALDI-QIT-TOF-MS. **(B,C)** GSTP WT, GSTP S184A, GSTP S184D, or empty vector was transfected into cells, and after 36 h, cells were stimulated with or without LPS (500 ng/ml) for 6 h. **(B)** Cytoplasmic and nuclear fractions were subjected to Western blot using Flag-GSTP and HMGB1 antibodies. **(C)** Cells were incubated with anti-Flag antibody and then incubated with Alexa flour488-conjugated secondary antibody (green). The nuclei were counterstained with DAPI (blue). The location of GSTP was observed under a confocal laser microscope. Scale bar: 10 µm. **(D,E)** Primary peritoneal macrophages **(D)** and RAW264.7 cells **(E)** were transfected with GSTP WT, GSTP S184A, GSTP S184D, or empty vector, and then were stimulated with or without LPS (500 ng/ml) for 18 h. HMGB1 level in the culture medium was determined by ELISA. **(F)** RAW264.7 cells were transfected with GSTP WT, GSTP S184D, GSTP S42A, GSTP S42D, GSTP Y198F, GSTP Y198D. After 36 h, cells were incubated with or without LPS (500 ng/ml) for 6 h. Nuclear and cytoplasmic fractions were analyzed by Western blot using Flag-GSTP and HMGB1 antibodies. **(G)** RAW264.7 cells transfected with GSTP WT, GSTP S184D, GSTP S42A, GSTP S42D, GSTP Y198F, GSTP Y198D were incubated with or without LPS (500 ng/ml) for 18 h, and then levels of HMGB1 in the culture medium were measured by ELISA. **(H)** HEK293 cells were transiently transfected with Flag-HMGB1, and cell extracts were incubated with wild-type His-GSTP or its mutants immobilized on Ni^2+^–IDA–agarose beads for 8 h, followed by immunoblotting with anti-His or anti-Flag antibody. **(I)** HEK293 cells were transfected with wild-type Flag-GSTP or its mutants and then incubated with His-tagged HMGB1. Data information: In **(B,F)**, data are presented as mean ± SD. *P<0.05; **P<0.01 versus corresponding nuclear group by unpaired Student’s *t*-test. ^#^*P*<0.05; ^##^*P*<0.01 versus corresponding cytoplasmic group by unpaired Student’s *t*-test. In **(D,E,G)**, data are presented as mean ± SD. **P<0.01; n.s., not significant (*P* > 0.05) versus corresponding LPS-treated group by unpaired Student’s *t*-test.

Protein post-translational modifications, such as phosphorylation is closely related with protein subcellular location (50). Some reports indicated that GSTP could be phosphorylated at Ser-42, Ser-184, Tyr-3, Tyr-7, or Tyr-198 in human glioblastoma cells (51–53). We next evaluated if phosphorylation was involved in GSTP nucleus translocation. RAW264.7 cells were transfected with Flag-GSTP and treated with LPS for the indicated period. The cell lysates were immunoprecipitated with Flag-antibody followed by immunoblotting analysis using p-Ser, p-Tyr, and p-Thr antibodies. The results showed that the level of serine phosphorylated GSTP increased within 15 min after LPS stimulation and slightly reduced at 30 min (Figure S4A in Supplementary Material). The tyrosine phosphorylated GSTP level was high in untreated cells and slightly increased from 15 to 30 min after LPS stimulation. No obvious threonine phosphorylated GSTP was detected (Figure S4A in Supplementary Material). MALDI-QIT-TOF-MS analysis showed that in unstimulated RAW264.7 cells, there were four phosphorylated residues including Tyr3, Tyr7, Ser42, and Tyr198 in GSTP, whereas five phosphorylated amino acids including Tyr3, Tyr7, Ser42, Tyr198, and Ser184 in GSTP could be observed in LPS-stimulated cells (Figure 5A). We noticed that LPS stimulation resulted in Ser184 phosphorylation and greatly raised the phosphorylated Ser42 and Tyr198 level (Figure 5A), but phosphorylation of Tyr3 and Tyr7 was not altered by LPS challenge.

In order to determine if the phosphorylation of Ser42, Ser184, and Tyr198 was involved in the effect of GSTP on HMGB1, six GSTP mutants were constructed, including GSTP-S42A (a Ser42 non-phosphorylatable mutant), GSTP-S42D (a Ser42 constant-phosphomimetic mutant), GSTP-S184A (a Ser184 non-phosphorylatable mutant), GSTP-S184D (a Ser184 constant-phosphomimetic mutant), GSTP-Y198F (a Tyr198 non-phosphorylatable mutant), as well as GSTP-Y198D (a Tyr198 constant-phosphomimetic mutant) (Figure S4B in Supplementary Material). The immunoblotting and immunofluorescence microscopy detection demonstrated that only GSTP-S184D entered the nucleus in LPS-untreated RAW264.7 cells, and LPS triggered GSTP (WT) but not GSTP-S184A to enter the nucleus (Figures 5B,C). Furthermore, the level of nuclear GSTP-S184D was higher than GSTP (WT) after LPS treatment (Figures 5B,C). As shown in Figures 5D,E and Figure S4C in Supplementary Material, GSTP-S184D performed stronger inhibitory effect on LPS-induced HMGB1 release than GSTP (WT) in primary peritoneal macrophages, and RAW 264.7 and THP-1 cells, but GSTP-S184A showed a smaller influence. Consistently, GSTP-S184D also showed higher efficiency than GSTP (WT) in preventing LPS-induced HMGB1 export from the nucleus to cytoplasm in RAW264.7 cells, while GSTP-S184A almost had no effect on that (Figure 5B). As LPS enhanced the phosphorylated Ser42 and Tyr198 in GSTP, we transfected GSTP mutants including GSTP-S42A, GSTP-S42D, GSTP-Y198F, GSTP-Y198D, and GSTP-S184D into RAW264.7 cells and then treated cells with LPS for 18 h. As shown in Figure 5F, there was almost no GSTP-S42A, GSTP-S42D, GSTP-Y198F, or GSTP-Y198D protein in the nucleus of LPS-unstimulated cells, and all GSTP mutant proteins appeared in the nucleus after LPS stimulation. Furthermore, all GSTP-S42A, GSTP-S42D, GSTP-Y198F, and GSTP-Y198D acted the same in suppressing LPS-induced HMGB1 cytoplasmic translocation and release in RAW264.7 cells (Figures 5F,G). Based on these results, it could be demonstrated that GSTP regulated LPS-induced HMGB1 translocation and release was independent of Ser42 and Tyr198 phosphorylation.

To confirm the importance of Ser184 phosphorylation of GSTP in the interaction of GSTP with HMGB1, His-tagged GSTP (WT), GSTP-S184D, and GSTP-S184A proteins were expressed in *E. coli* and purified. *In vitro* pull-down assay showed that GSTP-S184D displayed a stronger affinity to bind HMGB1 than GSTP (WT), but GSTP-S184A did not combine with HMGB1, indicating that Ser184 phosphorylation was necessary for the association between GSTP and HMGB1 (Figures 5H,I).

### cPKC Is the Upstream Kinase for GSTP Phosphorylation at Ser184

**Figure 6:**
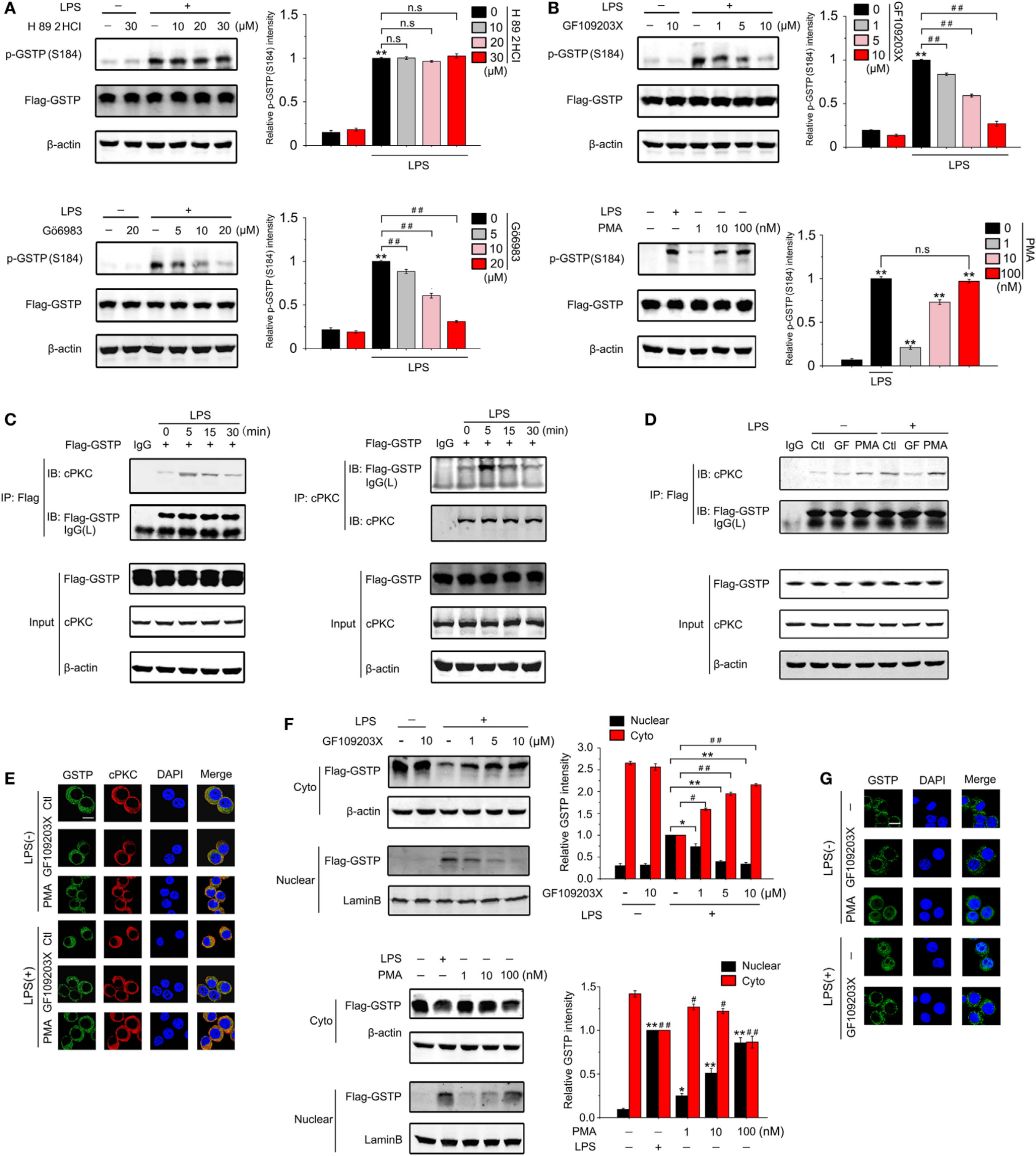
Ser184 of glutathione *S*-transferase Pi (GSTP) is phosphorylated by classic protein kinase C (cPKC) under lypopolysaccharide (LPS) stimulation. **(A,B)** RAW264.7 cells were transfected with Flag-GSTP, followed by pretreatment with or without H 89 2HCl, Gö6983, or GF109203X for 2 h, and then stimulated with LPS (500 ng/ml) or phorbol-12-myristate-13-acetate (PMA) for 30 min. Western blot analysis was performed by using anti-p-GSTP (S184) antibody. **(C)** RAW264.7 cells were transfected with Flag-GSTP. After 36 h, cells were incubated with LPS (500 ng/ml) for 0, 5, 15, or 30 min. Cell lysates were immunoprecipitated with anti-Flag antibody(left) or anti-cPKC (right) and then were immunoblot analyzed with anti-cPKC and anti-Flag antibodies. Whole cell lysates were subjected to immunoblotting with anti-Flag, anti-cPKC and anti-β-actin antibodies. **(D)** RAW264.7 cells were transfected with Flag-GSTP and pre-treated with or without GF109203X (10 µM) for 2 h, followed by treatment of PMA (100 nM), LPS (500 ng/ml), or both for 5 min. Cell lysates were immunoprecipitated with anti-Flag antibody and then were immunoblot analyzed by using anti-Flag, anti-cPKC, and anti-β-actin antibodies. **(E)** RAW264.7 cells were pretreated with or without GF109203X (10 µM) for 2 h, and then were stimulated with LPS (500 ng/ml), PMA (100 nM), or both for 5 min. After that, cells were incubated with rabbit anti-GSTP and mouse anti-cPKC antibodies, and then, were incubated with Alexa flour488-conjugated anti-rabbit (green) and Alexa flour555-conjugated anti-mouse (red) secondary antibodies. The nuclei were counterstained with DAPI (blue). The complex of GSTP and cPKC was observed by confocal laser microscopy. Scale bar: 10 µm. **(F)** RAW264.7 cells were transfected with Flag-GSTP, followed by pretreatment with or without indicated concentrations of GF109203X for 2 h and stimulation with LPS (500 ng/ml) (upper) or PMA (100 nM) (lower) for 6 h. Nuclear and cytoplasmic fractions were analyzed by Western blot using anti-Flag antibody (upper). **(G)** RAW264.7 cells were pretreated with or without GF109203X (10 µM) for 2 h followed by stimulating with LPS (500 ng/ml) or PMA (100 nM) for 6 h, and then microscopic images were captured to show GSTP (green) and DAPI (blue) in cells. Scale bar: 10 µm. The blots were representative of three independent experiments. Data information: in **(A,B)**, data are presented as mean ± SD. ^##^*P*<0.01; n.s., not significant (*P* > 0.05), versus corresponding LPS-treated group by unpaired Student’s *t*-test. In **(F)**, *P<0.05; **P<0.01 versus corresponding LPS-treated nuclear group by unpaired Student’s *t*-test. ^#^*P*<0.05; ^##^*P*<0.01 versus corresponding LPS-treated cytoplasmic group by unpaired Student’s *t*-test (lower). *P<0.05; **P<0.01 versus untreated nuclear group by unpaired Student’s *t*-test. ^#^*P*<0.05; ^##^*P*<0.01 versus untreated cytoplasmic group by unpaired Student’s *t*-test.

Lo et al reported that cAMP-dependent protein kinase (PKA) and protein kinase C (PKC) activation resulted in phosphorylation of GSTP in MGR3 human glioblastoma cells (51). To determine which kinase was involved in phosphorylation of GSTP at Ser184 in LPS-stimulated macrophages, we treated RAW264.7 cells with PKA or PKC inhibitor, respectively. As shown in Figures 6A,B, H 89 2HCl (a PKA inhibitor) did not affect GSTP Ser184 phosphorylation, whereas Gö6983 (a broad-spectrum PKC inhibitor) and GF109203X (a selective inhibitor of classic PKC, cPKC) apparently attenuated LPS-stimulated Ser184 phosphorylation. PMA is a surrogate of diacylglycerol, that can activate cPKC and novel PKC (nPKC) isoenzymes. GSTP Ser184 phosphorylation increased, when we treated RAW264.7 cells with PMA, which further indicated the involvement of cPKC in GSTP Ser184 phosphorylation. Coimmunoprecipitation experiments showed that when RAW264.7 cells were transfected with Flag-GSTP, the complex of cPKC/Flag-GSTP increased to the maximum at 5 min after LPS treatment and then maintained a high level from 15 to 30 min (Figure 6C). Moreover, both coimmunoprecipitation and confocal microscopy studies showed that PMA promoted the interaction between cPKC and GSTP in LPS-unstimulated cells, while GF109203X inhibited the binding of cPKC to GSTP in LPS-stimulated cells (Figures 6D,E). These results strongly suggested that cPKC acted as the upstream kinase of GSTP to phosphorylate Ser184 of GSTP in macrophages. Consistent with the result that Ser184 phosphorylation was essential for GSTP to enter nucleus, both immunoblotting and confocal microscopy detection demonstrated that GF109203X inhibited LPS-triggered GSTP nucleus translocation, and PMA promoted GSTP to enter the nucleus in LPS-untreated RAW264.7 cells (Figures 6F,G). H 89 2HCl had no effect on the translocation of GSTP (Figure S5 in Supplementary Material).

### Nuclear GSTP Inhibits HMGB1 Phosphorylation and Increases its DNA-Binding Affinity

**Figure 7:**
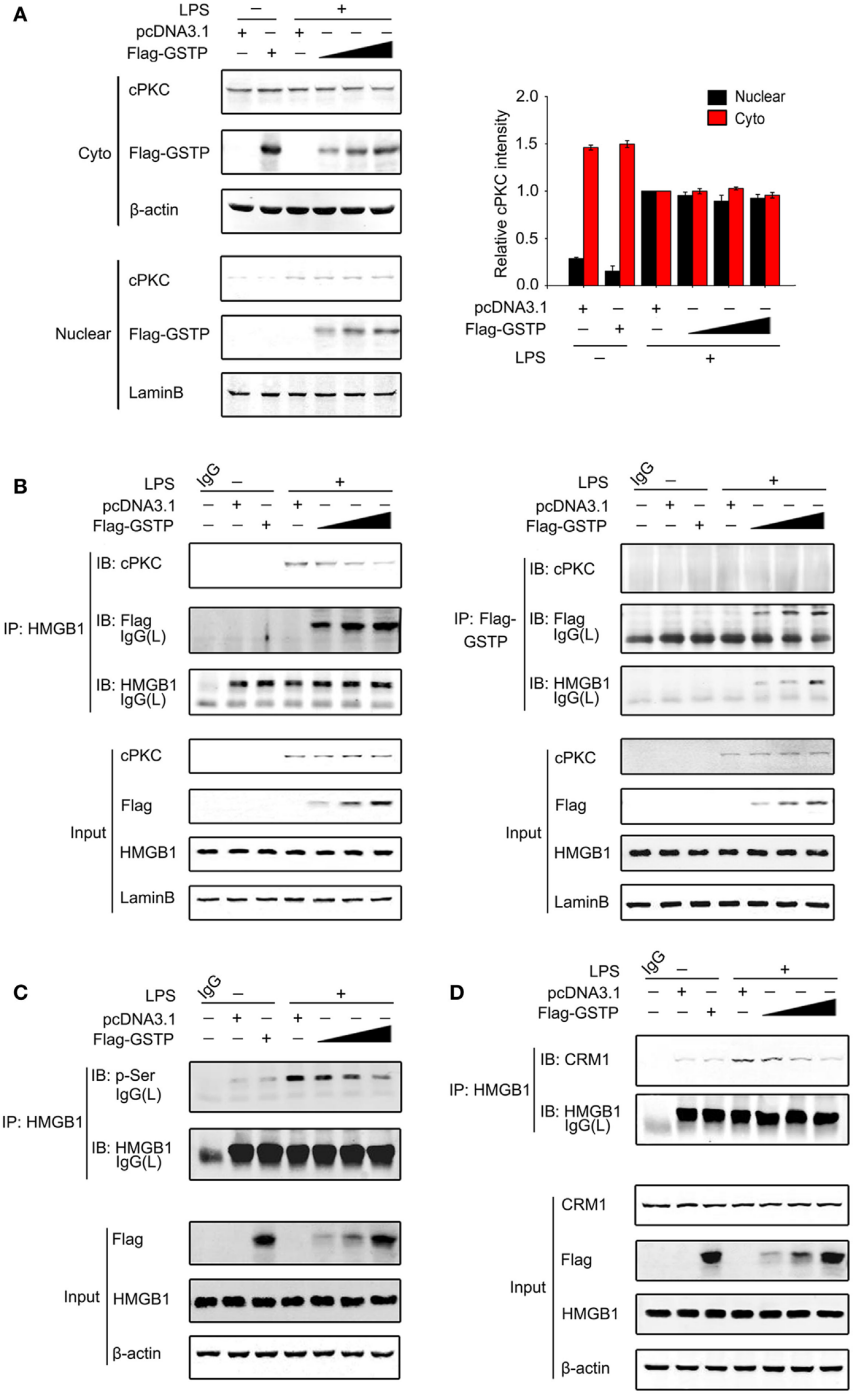
Nuclear glutathione *S*-transferase Pi (GSTP) inhibits the interaction between classic protein kinase C (cPKC) and high mobility group box-1 protein (HMGB1). **(A,B)** Flag-GSTP (0.5, 1, or 2 µg) or empty vector was transfected into RAW264.7 cells. After 36 h, cells were incubated with lypopolysaccharide (LPS; 500 ng/ml) for 20 min. **(A)** Nuclear and cytoplasmic fractions were subjected to immunoblot analysis with anti-cPKC or anti-Flag antibody. **(B)** Nuclear extracts were subjected to immunoprecipitation with anti-HMGB1 or anti-Flag antibody and then were immunoblot analyzed with anti-Flag and anti-HMGB1 antibodies. Whole nuclear lysates were immunoblot analyzed by using anti-cPKC, anti-Flag, anti-HMGB1, or anti-lamin B antibody, respectively. **(C,D)** Flag-GSTP (0.5, 1, or 2 µg) or empty vector was transfected into RAW264.7 cells. **(C)** Cells were incubated with LPS (500 ng/ml) for 4 h. Cell lysates were immunoprecipitated using HMGB1 antibody and analyzed by immunoblotting with p-Ser antibody. Whole cell lysates were subjected to immunoblotting with Flag-GSTP, HMGB1 and β-actin antibodies. **(D)** Cells were treated with LPS (500 ng/ml) for 6 h. Cell lysates were immunoprecipitated with anti-HMGB1 antibody and analyzed by immunoblotting using HMGB1 and chromosomal region maintenance 1 antibodies. The blots were representative of three independent experiments.

Classic protein kinase C has been reported to enter the nucleus and act as an upstream kinase of HMGB1 in LPS-stimulated macrophages and promotes HMGB1 to export from the nucleus to cytoplasm (29, 30). As shown in Figure 7A, cPKC entered nucleus of RAW264.7 cells 20 min after LPS treatment, which was not prevented by overexpression of GSTP. However, the interaction between cPKC and HMGB1 and the HMGB1 phosphorylation were markedly suppressed by overexpression of GSTP (Figures 7B,C). These results indicated that GSTP hampered the association of HMGB1 with cPKC, but not cPKC translocation. Previous reports demonstrated that HMGB1 interacted with CRM1 for active export from nuclear (25). Our coimmunoprecipitation study showed that LPS enhanced CRM1/HMGB1 complex formation (Figure 7D), whereas, overexpression of GSTP substantially attenuated interaction between HMGB1 and CRM1. Altogether these results indicated that nuclear GSTP restrained HMGB1 cytoplasm translocation through preventing the interaction between cPKC and HMGB1 and attenuating the interaction between HMGB1 and CRM1.

## Discussion

Since the pathological process of sepsis-induced multiple organ dysfunction remains incompletely understood, the treatment of sepsis has not significantly changed over the past 40 years (54). In the present study, we provide the novel evidence that *Gstp*^−/−^ mice showed both the notable higher mortality rate and serum HMGB1 level, as well as severer septic pulmonary injury compared with wild-type mice after CLP surgery. These findings reveal for the first time that endogenous GSTP negatively regulates excessive inflammatory response *in vivo* and inhibits sepsis-related organ dysfunction and death. Another novel result in our study is that the recombinant GSTP protein significantly increases the survival rate of CLP mouse and decreases the level of mouse serum HMGB1, which strongly suggests that recombinant GSTP protein is potential for sepsis therapy.

The biggest obstacle to the discovery of therapeutics for sepsis is its diverse etiology among patients (55), which have prompted researchers to study the molecular mechanism of sepsis progression based on systemic inflammatory responses. It has been well documented that sepsis occurs because of over-activated immune responses which include intractable inflammatory responses associated with imbalanced cytokine production (18). The increase of serum HMGB1 appears to be a key lethal factor in sepsis, and the control of HMGB1 is crucial in the management of sepsis (56, 57). Previous researches have shown that HMGB1 inhibitor BoxA reversed lethality of established sepsis, decreased sepsis-induced organ injury, prevented HMGB-induced leukocyte recruit, diminished CTL-induced liver disease in HBV transgenic mice (58–61). Our findings revealed that *Gstp* gene knocked caused a significant increase in mouse serum HMGB1 level, suggesting that GSTP might function as a negative regulator in controlling HMGB1 release in sepsis.

Lypopolysaccharide stimulation may lead to HMGB1 to export from the nucleus to cytoplasm where it condenses into secretory lysosomes and is actively secreted from monocytes and macrophages (62). Our *in vitro* studies on primary peritoneal macrophages from *Gstp*^−/−^ mice and wild-type mice, and cell lines of RAW264.7 and THP-1 indicated that GSTP inhibited LPS-induced HMGB1 release through preventing its translocation from the nucleus to cytoplasm. GSTs are generally grouped into three classes based on their subcellular localization: membrane-bound microsomal, mitochondrial, and cytoplasmic (63). GSTP belongs to a member of cytosolic GST family and so far, most reports about GSTP have described the function of cytosolic GSTP (64, 65). Although Goto et al. showed the possible role of nuclear GSTpi in the acquisition of resistance to anticancer drugs by cancer cells (66, 67), it is unclear how GSTP translocated from the cytoplasm to nucleus and if in the macrophage, cytosolic GSTP could also enter into the nucleus. In the present study, we found that GSTP prevented nuclear-cytoplasmic translocation of HMGB1, which strongly suggested that GSTP functioned in nucleus of the macrophage. Interestingly, our results demonstrated that cytosolic GSTP entered nucleus after the macrophage being stimulated by LPS.

When probing how LPS triggered the nucleus transportation of GSTP, we found that the phosphorylation modification of GSTP was necessary in this process. Phosphorylation is the common modification of the proteins, which often alters various properties of proteins, including subcellular localization. GSTP has been reported to be phosphorylated at four residues including Ser-42, Ser-184, Tyr-7, and Tyr-198 in human glioma cells (51–53). In these papers, GSTP phosphorylation is linked with its catalytic activity and drug resistance of tumor cells rather than the translocation of GSTP from the cytoplasm to nucleus. Our data from MALDI-QIT-TOF-MS indicated that only Ser184 phosphorylation was dependent on LPS stimulation. Using the mutant GSTP-S184A and GSTP-S184D plasmids, we found that Ser184 phosphorylation is essential for GSTP to enter the nucleus and to inhibit cytoplasmic translocation of HMGB1 in LPS-activated macrophages, while the phosphorylation of GSTP at Ser42, Tyr3, Tyr7, and Tyr198 is unnecessary. GSTM is different in approximate 70% sequence of amino acid from GSTP, including 184-residue. GSTM could not enter the nucleus of RAW264.7 cells under LPS stimulation, further confirming the importance of Ser184 phosphorylation for GSTP to enter the nucleus. PKC is a family of isoenzymes, including cPKC, nPKC, and atypical PKC (68). Although both PKC and PKA have been reported to be the upstream kinase for Ser184 of GSTP in MGR3 human glioblastoma cells (51), our study indicated that only cPKC interacted with GSTP and mediated LPS-stimulated GSTP phosphorylation. It is not surprised since LPS-induced cPKC activation has been demonstrated in the recent reports (30, 69). It has been documented that nuclear-cytoplasmic translocation of HMGB1 in LPS-treated macrophages depended on its phosphorylation by cPKC or calcium/calmodulin-dependent protein kinase IV (CaMKIV) (29–31, 70–72). It is worth noting that, nuclear GSTP inhibited the association between HMGB1 and cPKC and reduced cPKC-mediated HMGB1 phosphorylation through interacting with HMGB1 in response to LPS stimulation. In addition, GSTP also prevented the binding of HMGB1 to CRM1 suggesting GSTP suppressed CRM1-mediated HMGB1 nuclear export. Although GSTP is an enzyme to catalyze protein S-glutathionylation under oxidative stress (21, 22), we found that GSTP regulated HMGB1 release in an enzyme activity independent manner. More interestingly, the present study showed that cPKC acted as the upstream kinase of both GSTP and HMGB1, phosphorylate both of them in macrophages under LPS stimulation. While it phosphorylated GSTP in the cytoplasm, cPKC also entered into the nucleus to phosphorylate HMGB1. Phosphorylation of GSTP did not prevent the translocation of cPKC into the nucleus, but entered the nucleus to prevent HMGB1/cPKC association. Thus, the activation of cPKC played two contrary roles during infection. cPKC activated pro-inflammatory signal pathways through promoting HMGB1 release on the one hand, on the other hand, it inhibited HMGB1 release by phosphorylating GSTP. The new function of GSTP we found in the present study is that it acts as a negative regulator in the pro-inflammatory signal transduction and in the pathogenesis of sepsis. We also provide the novel evidence that the phosphorylation by cPKC is an important modification for GSTP to translocate from cytoplasm to nucleus and to antagonize the effect of cPKC on HMGB1. It is not surprised that if we raised cytosolic GSTP level through GSTP plasmid transfection or treated mice with GSTP protein, the excessive inflammatory responses and mortality rate of CLP-induced sepsis were significantly reduced.

**Figure 8:**
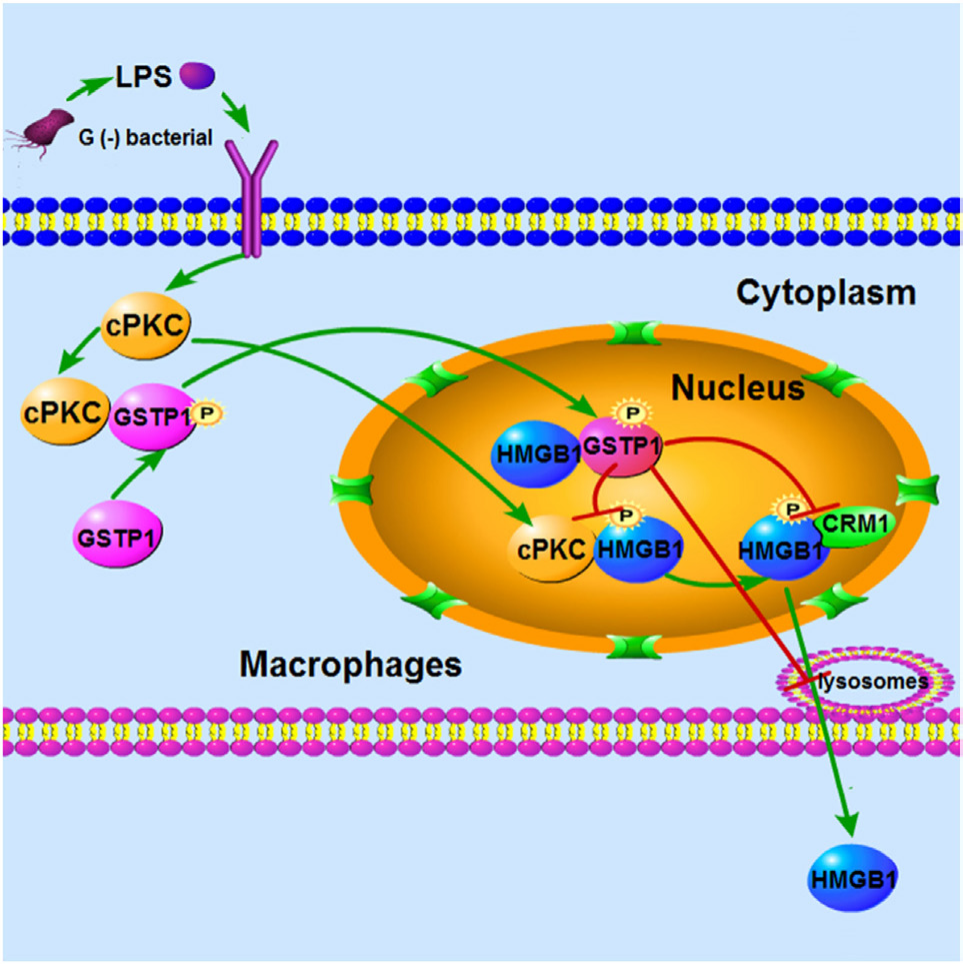
Conceptual relationships between glutathione *S*-transferase Pi (GSTP), classic protein kinase C (cPKC), and high mobility group box-1 protein (HMGB1) in lypopolysaccharide (LPS)-induced macrophages. LPS stimulation induced cPKC activation, and the activated cPKC leads to GSTP phosphorylation and nuclear translocation. Nuclear GSTP binds to HMGB1 and then inhibits it transfer to the plasma and release from macrophages.

In conclusion, the *in vivo* work on *Gstp*^−/−^ mice fully demonstrates that GSTP acts as a negative regulator in sepsis pathophysiology, and recombinant GSTP protein treatment to mice may significantly improve survival in mouse CLP sepsis model. In addition, our *in vitro* studies indicate a novel mechanism, that is GSTP can be phosphorylated at Ser184 and enter the nucleus under LPS stimulation, and GSTP interacted with HMGB1 to inhibit cPKC-mediated phosphorylation of HMGB1 and association between HMGB1 and CRM1 in the nucleus. Consequently, GSTP1 prevents HMGB1 cytoplasmic translocation and release from macrophages (Figure 8). cPKC is the upstream kinase of both GSTP and HMGB1, which plays the opposite roles in HMGB1 release through phosphorylating GSTP or HMGB1, respectively. To sum up, our findings strongly suggest that GSTP is a potential target for treating sepsis.

## Ethics Statement

This study was carried out in accordance with the recommendations of on ARRIVE and recommendations from an NIH-sponsored workshop regarding experimental design and reporting standards. These experiments had been approved by Medical Laboratory Animal Research Institute of Medical Sciences China [Permit Number: SYXK (Jing) 2014-0004]. All mice were handled in full compliance with the animal welfare regulations and maintained according to the standard protocols.

## Author Contributions

Conceived and designed the experiments: ZY, LL, YZ, and PC. Performed the experiments: YZ, XC, YY, JW, WY, PB, and LS. Analyzed the data: YZ, XC, LL, ZY, and PC. Wrote the paper: LL, YZ, and ZY.

## Conflict of Interest Statement

The authors declare that the research was conducted in the absence of any commercial or financial relationships that could be construed as a potential conflict of interest.
