# Supplementary material for: Glutathione S-Transferase Pi Prevents Sepsis-Related High Mobility Group Box-1 Protein Translocation and Release
Source: Front Immunol. 2018 Feb 19;9:268. doi: 10.3389/fimmu.2018.00268 (PMC5827551; doi:10.3389/fimmu.2018.00268)
Supplement: Supplementary file 1 [file Presentation_1.pdf]

Supplementary Material

GSTP prevents sepsis related HMGB1 translocation and release

Yi Zhou,[1] Xiang Cao,[1] Yang Yang,[2,3] Jing Wang,[1] Weidong Yang,[1] Peiling Ben,[1] Lei Shen,[1] Peng Cao[3]*, Lan Luo,[2,]* and Zhimin Yin[1,]*

1 Jiangsu Province Key Laboratory for Molecular and Medical Biotechnology, College of Life Science, Nanjing Normal University, Nanjing, 210046, Jiangsu, P R China

2 State Key Laboratory of Pharmaceutical Biotechnology, School of Life Sciences, Nanjing University, Nanjing 210093, Jiangsu, P R China

3 Laboratory of Cellular and Molecular Biology, Jiangsu Province Institute of Traditional Chinese Medicine, 100#, Shizi Street, Hongshan Road, Nanjing 210028, Jiangsu, China

*Correspondence: yinzhimin@njnu.edu.cn (Z.M.Y), lanluo@nju.edu.cn (L.L), pcao79@yahoo.com (P.C)

Supplementary Figures

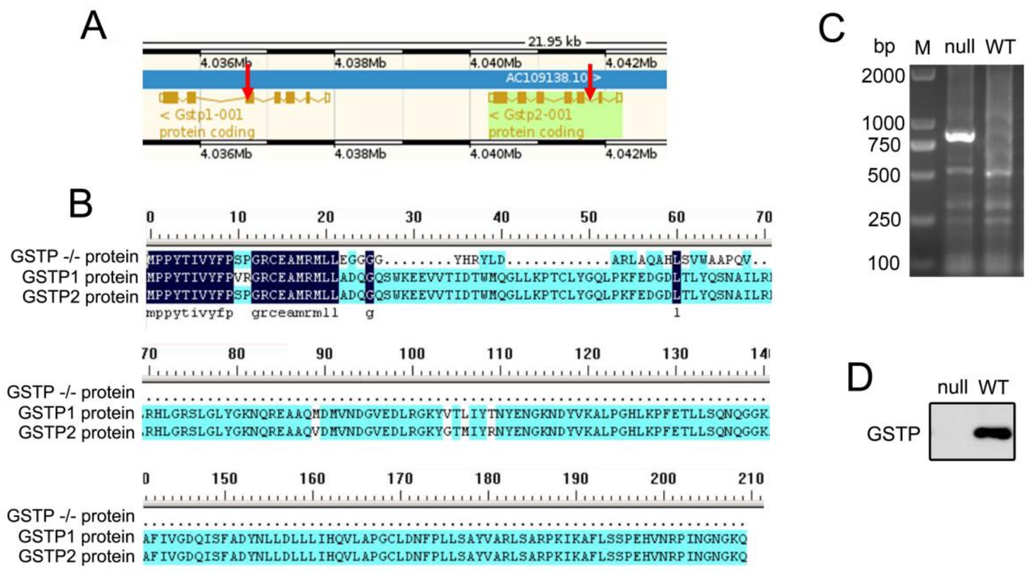

Figure S1. Construction and identification of *Gstp* knockout mice (Related to Figure 1,2).

A. *Gstp1* and *Gstp2* genes lie adjacent to one another on chromosome 19, the red arrows showed the cutting position of target gene by CRISPR/Cas9 technology.
B. The GSTP protein sequences of knockout mice and wild type mice were displayed by DNAman.
C. The DNA of *Gstp* knockout (null) mice and wild type mice were extracted and PCR, and the PCR products were identified using agarose gel electrophoresis.
D. The protein of *Gstp* knockout (null) mice and wild type mice were extracted and detected by Western blot analysis.

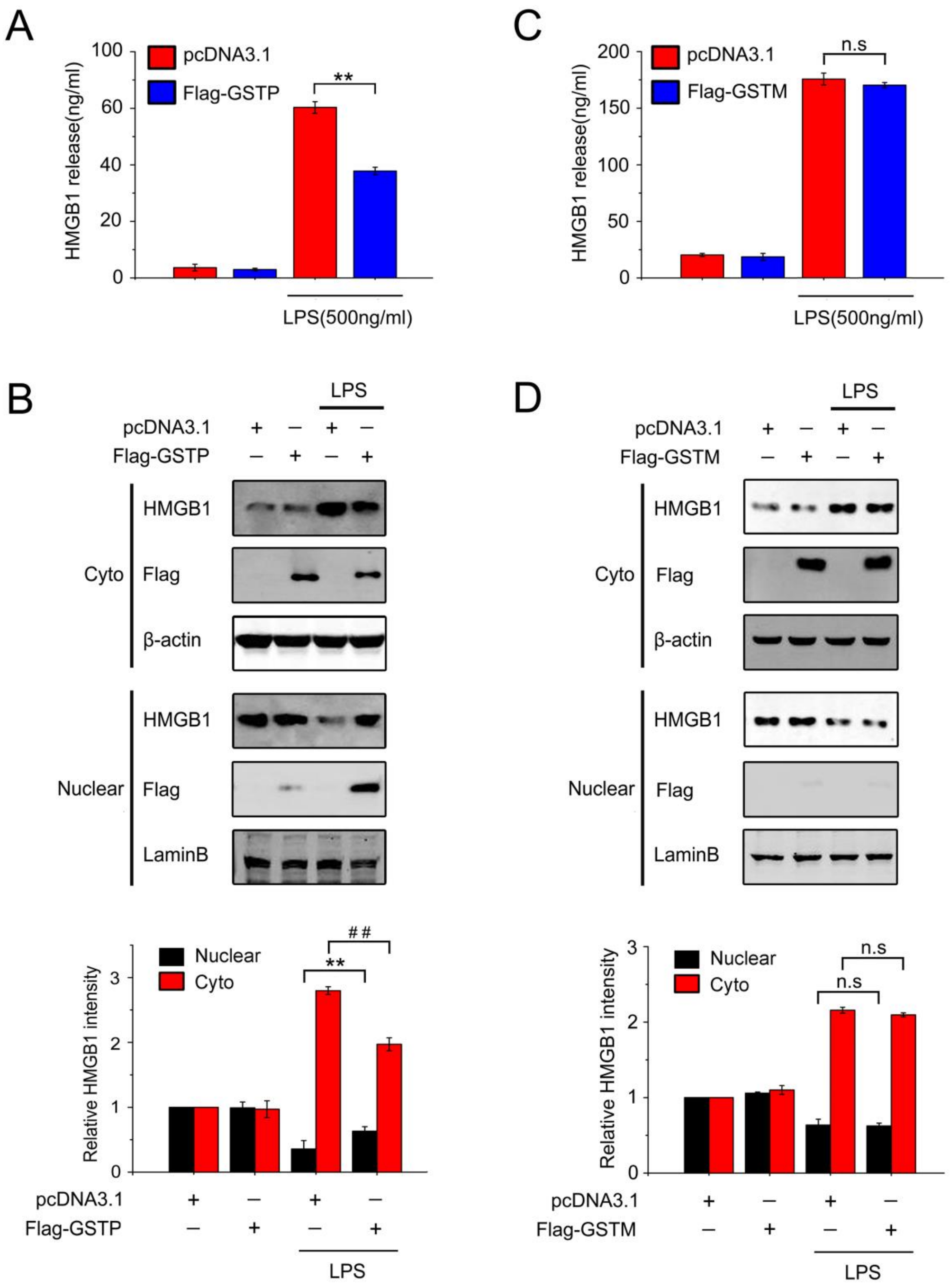

Figure S2. Over-expressed GSTP, but not GSTM, influences LPS-induced release

and translocation of HMGB1 (Related to Figure 2).

A, B. THP-1 cells were transiently transfected with Flag-GSTP or empty vector for 36 h followed by stimulation with or without LPS (500ng/ml). (A) Eighteen hours after LPS treatment, the levels of HMGB1 in the culture medium were measured by ELISA. (B) Sixteen hours after LPS treatment, nuclear and cytoplasmic fractions were extracted and subjected to Western blot for detecting cellular HMGB1 and Flag-GSTP. Equal loading protein was determined by the expression of nuclear lamin B and β-actin.

C, D. RAW264.7 cells were transiently transfected with Flag-GSTM or empty vector followed by stimulation with LPS (500ng/ml). (C) Eighteen hours after LPS treatment, the levels of HMGB1 in the culture medium were measured by ELISA. (D) Sixteen hours after LPS treatment, nuclear and cytoplasmic fractions were extracted and subjected to Western blot for detecting cellular HMGB1 and Flag-GSTM. Data shown were representative of three independent experiments. ***P*＜0.01 compared with the cells transfected with the empty vector.

Data information: In (A, C), data are presented as mean ± SD. $^{\#\#}$, P＜0.01; n.s, not significant (P > 0.05), versus corresponding LPS-treated group by unpaired Student's *t* test. In (B, D), data are presented as mean ± SD. **, P＜0.01 versus corresponding LPS-treated nuclear group by unpaired Student's *t* test. $^{\#\#}$, P＜0.01 versus corresponding LPS-treated cytoplasmic group by unpaired Student's *t* test. n.s, not significant (P > 0.05).

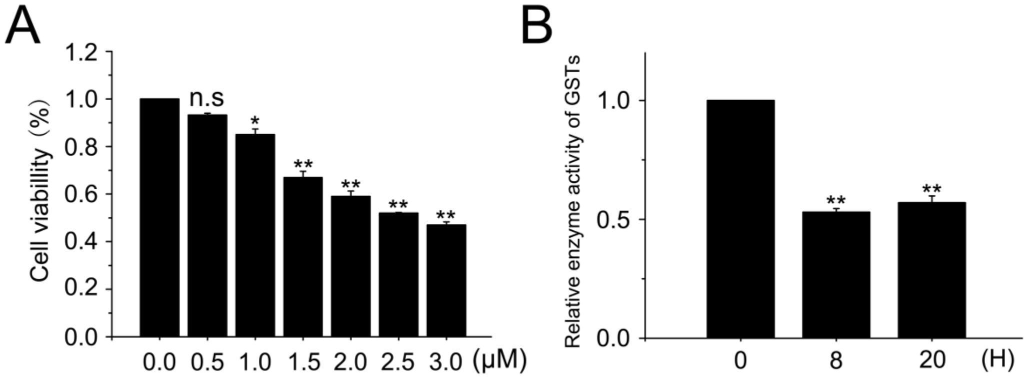

Figure S3. NBDHEX inhibits GSTP enzymatic activity (Related to Figure 3).

A. RAW264.7 cells were incubated with indicated concentration of NBDHEX for 48 h. Cell viability was detected by CCK8 assay.

B. RAW264.7 cells were exposed to NBDHEX at 0.5μM concentration for indicated time. Then cells were harvested, and the GSTP enzymatic activity were determined by using glutathione S-transferase (GSH-ST) kit. Data shown were representative of three independent experiments.

Data information: In (A-B), data are presented as mean ± SD. *, P＜0.05; **, P＜0.01; n.s, not significant (P > 0.05), versus group untreated with NBDHEX by unpaired Student's *t* test.

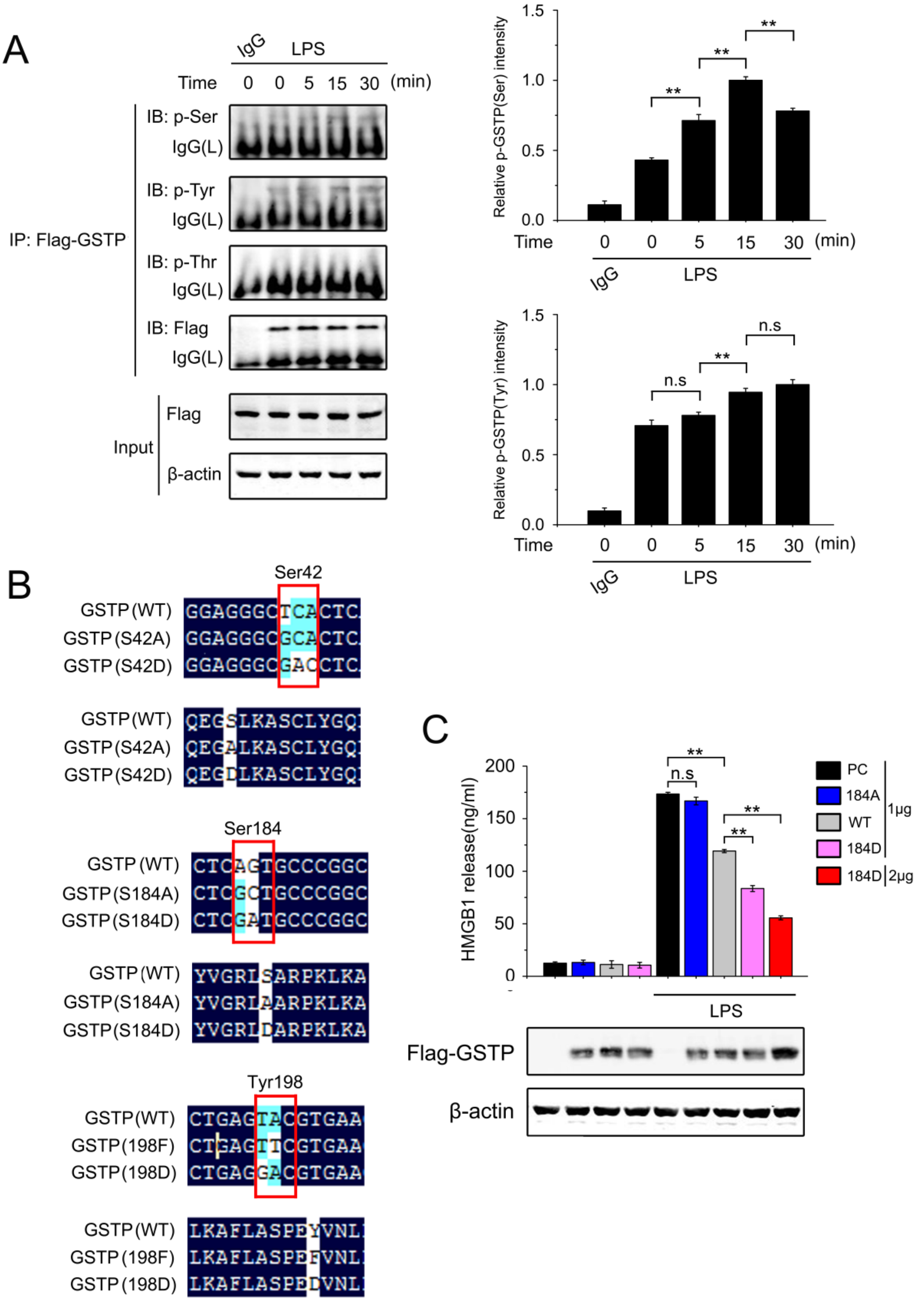

Figure S4. Effect of S184 phosphorylation of GSTP on HMGB1 release (Related to Figure 5).

A. RAW264.7 cells were transfected with Flag-GSTP. After 36 h, the cells were incubated with LPS (500ng/ml) for 0, 5, 15 or 30 min. Cell lysates were subjected to immunoprecipitated with anti-Flag antibody and immunoblot analysed with anti-p-Ser, anti-p-Tyr, anti-p-Thr and anti-Flag antibodies. The whole cell lysates were subjected

to immunoblotting with anti-Flag and anti-β-actin antibodies.
B. Six GSTP mutants were constructed: GSTP-S42A (serine-42 was mutated to alanine, a Ser42 non-phosphorylatable mutant), GSTP-S42D (serine-42 was mutated to aspartate, a Ser42 constant-phosphomimetic mutant), GSTP-S184A (serine-184 was mutated to alanine, a Ser184 non-phosphorylatable mutant), GSTP-S184D (serine-184 was mutated to aspartate, a Ser184 constant-phosphomimetic mutant), GSTP-Y198F (tyrosine-198 was mutated to phenylalanine, a Tyr198 non-phosphorylatable mutant) and GSTP-Y198D (tyrosine-198 was mutated to aspartate, a Tyr198 constant-phosphomimetic mutant). Sequencing results of DNA (upper panel) and protein (bottom panel) were displayed.
C. THP-1 cells were transiently transfected with GSTP WT (1μg), GSTP S184A (1μg), GSTP S184D (1μg or 2μg) or pcDNA3.1. Thirty-six hours after transfection, cells were stimulated with or without LPS (500ng/ml) for 18 h, then HMGB1 levels in the culture medium were determined by ELISA .
Data information: In (A, C), data are presented as mean ± SD. **, P＜0.01; n.s, not significant (P > 0.05), versus corresponding LPS-treated group by unpaired Student's t test.

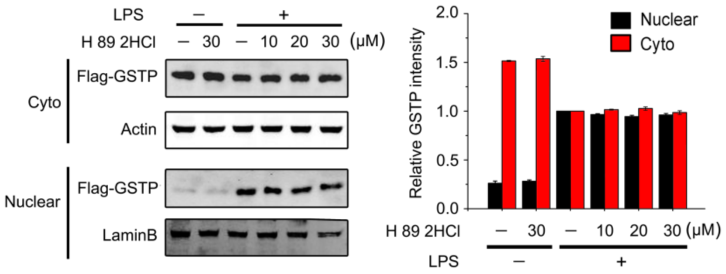

Figure S5. Effect of PKA inhibitor on LPS-induced nuclear translocation of GSTP (Related to Figure 6).
RAW264.7 cells were transiently transfected with Flag-GSTP followed by incubation with or without indicated concentrations of H 89 2HCl for 2 h, then cells were stimulated with LPS (500ng/ml) for 6 h. Nuclear and cytoplasmic fractions were analyzed for detection of Flag-GSTP by Western blot. The blots were representative of three independent experiments.
